# Nomograms based on pre-operative parametric for prediction of short-term mortality in acute myocardial infarction patients treated invasively

**DOI:** 10.18632/aging.202230

**Published:** 2020-12-11

**Authors:** Qingjie Wang, Wei Qian, Zhiqin Sun, Wenwu Zhu, Yu Liu, Xin Chen, Yuan Ji, Ling Sun

**Affiliations:** 1 Department of Cardiology, The Affiliated Changzhou No. 2 People’s Hospital of Nanjing Medical University, Changzhou, Jiangsu, China; 2 Department of Pediatrics, The Affiliated Wuxi Children’s Hospital of Nanjing Medical University, Wuxi, Jiangsu, China; 3 School of Clinical Medicine, The Affiliated Changzhou No. 2 People’s Hospital of Nanjing Medical University, Changzhou, Jiangsu, China; 4 Section of Pacing and Electrophysiology, Division of Cardiology, The First Affiliated Hospital of Nanjing Medical University, Nanjing, Jiangsu, China

**Keywords:** acute myocardial infarction, mortality, nomogram, coronary angiography, percutaneous coronary intervention

## Abstract

Objective Our aim was to develop and independently validate nomograms to predict short-term mortality in acute myocardial infarction (AMI) patients.

Results There were 1229 AMI patients enrolled in this study. In the training cohort (n=534), 69 deaths occurred during a median follow-up period of 375 days. The C-index for 1-year mortality in the training group and the validation cohort was 0.826 (95%CI: 0.780 - 0.872) and 0.775 (95%CI: 0.695 - 0.855), respectively. Integrated Discrimination Improvement (IDI) and net reclassification improvement (NRI) also showed a significant improvement in the accuracy of the new model compared with the Global Registry of Acute Coronary Events (GRACE) risk score. Furthermore, C-index of the prospective cohort (n=309) achieved 0.817 (95%CI: 0.754 - 0.880) for 30-day mortality and 0.790 (95%CI: 0.718 - 0.863) for 1-year mortality.

Conclusions Collectively, our simple-to-use nomogram effectively predicts short-term mortality in AMI patients.

Methods AMI patients who had undergone invasive intervention between January 2013 and Jan 2018 were enrolled. Cox regression analysis was used on the training cohort to develop nomograms for predicting 30-day and 1-year mortality. Model performance was then evaluated in the validation cohort and another independent prospective cohort.

## INTRODUCTION

The clinical manifestations of patients with acute myocardial infarction (AMI) are different than other forms of cardiovascular disease and may include non-fatal ischemic events, and immediate and long-term risk of death [1]. Therefore, early risk stratification is essential for clinicians to choose the intensity of treatment by a risk assessment of individual patients [2].

Simple and well-validated tools play a pivotal role in predicting future events in the clinical practice, especially when deciding on the primary prevention and treatment for AMI patients. For example, the Global Registry of Acute Coronary Events (GRACE) risk score [3] is a reliable method for predicting adverse clinical events, including mortality and other major adverse cardiovascular events (MACE), improving prognosis and risk reclassification. However, little progress has been made to enhance the effectiveness of prognostic tools by combining new cardiac risk factors.

The primary purpose of this study was to develop and verify a robust prognostic model named Changzhou-AMI model (C-AMI), in predicting the 30-day and 1-year mortality of AMI patients. Our nomogram showed a potential advantage of predictive performance as compared with the GRACE risk score.

## RESULTS

### Clinical characteristics of patients

The clinical characteristics of the training cohort and the validation cohort are shown in Table 1. The mean age of 534 patients (69.5% male) was 68.3 ± 13.9 years in the training cohort, whereas the mean age of 386 patients (74.4% male) was 65.6 ± 13.3 years in the validation cohort. The 386 individuals in the validation cohort were slightly younger, with a lower prevalence of hypertension, diabetes and greater prevalence of smoking and alcohol intake. In the training group, there was a higher prevalence of patients with ST-segment elevation myocardial infarction (STEMI), and more patients received PCI therapy. The median follow-up was 375 days (IQR: 371 - 402) for the training cohort. And the median follow-up for the validation cohort was 390 days (IQR: 376 - 406). There were 69 deaths occurred in the training cohort and 32 deaths occurred in the validation cohort, respectively.

**Table 1:** Basic clinical and procedural characteristics.

**Variables**	**Total sample (n = 920)**	**Derivation cohort (n = 534)**	**Validation cohort (n = 386)**	***P* value**
**Demographics**				
Male, n (%)	658(71.5)	371(69.5)	287(74.4)	0.123
Age, y	67.18±13.70	68.33±13.86	65.59±13.29	0.003
20-29	4 (0.4)	3 (0.6)	1 (0.3)	
30-39	26 (2.8)	10 (1.9)	16 (4.1)	
40-49	105 (11.4)	68 (12.7)	37 (9.6)	
50-59	144 (15.7)	69 (12.9)	75 (19.4)	
60-69	228 (24.8)	113 (21.2)	115 (29.8)	
70-79	249 (27.1)	158 (29.6)	91 (23.6)	
80-89	154 (16.7)	106 (19.9)	48 (12.4)	
≥90	10 (1.1)	7 (1.3)	3 (0.8)	
SBP, mmHg	130.23±24.47	134.56±26.27	124.25±20.30	<0.001
DBP, mmHg	79.45±17.25	78.22±16.37	81.16±18.29	0.011
HR, bpm	81.98±17.26	82.26±17.80	81.60±16.49	0.566
**History Variables, n (%)**				
Hypertension	601(65.3)	381(71.3)	220(57.0)	<0.001
Diabetes mellitus	244(26.5)	156(29.2)	88(22.8)	0.036
Current smoker	460(50)	251(47.0)	209(54.1)	0.038
Alcohol intake	111(12.1)	52(9.7)	59(15.3)	0.014
**Laboratory findings**				
WBC, 10^9^/L	9.57±3.69	9.77±3.86	9.29±3.44	0.051
Neutrophil ratio (%)	76.32±11.45	77.80±9.88	74.28±13.07	<0.001
Hemoglobin, g/L	131.85±21.05	129.69±20.30	134.84±19.33	<0.001
Serum creatinine, mmol/L	95.15±56.51	107.23±66.69	78.43±31.35	<0.001
eGFR, mL/min/1.73m^2^	69.69±28.13	61.60±26.30	80.90±26.73	<0.001
HDL-C, mmol/L	1.18±0.39	1.23±0.39	1.12±0.38	<0.001
LDL-C, mmol/L	2.44±0.81	2.41±0.77	2.49±0.86	0.157
BNP	2160(431,4402)	2225(491,4313)	1769(386,4431)	0.26
Troponin I	2.01(0.52,8.04)	1.51(0.48,4.51)	3.83(0.62,14.98)	<0.001
**Outcomes**				
30-day mortality	70 (7.6)	49 (9.2)	21 (5.4)	0.047
1-year mortality	101 (11.0)	69 (12.9)	32 (8.3)	0.035
**Angiographic and Procedural Characteristics**				
STEMI	752(81.7)	465(87.1)	287(74.4)	<0.001
Number of Stent				0.005
0 (CAG only)	215(23.4)	107(20.0)	108(28.0)	
1	588(63.9)	345(64.6)	243(63.0)	
2	71(7.7)	42(7.9)	29(7.5)	
3	46(5.0)	40(7.5)	6(1.6)	
PCI	708(77.0)	427(80.0)	278(72.0)	0.005

Data are expressed as mean ± SD, n (%) or median (IQR). Abbreviations: SBP: systolic blood pressure; DBP: diastolic blood pressure; HR: heart rate; WBC: white blood cell, eGFR: estimated glomerular filtration rate (mL/min/1.73m^2^), HDL-C: High-density lipoprotein cholesterol, LDL-C: Low-density lipoprotein cholesterol, BNP: B-type natriuretic peptide, TNI: troponin I, STEMI: ST segment elevation myocardial infarction, PCI: percutaneous coronary intervention.

### Nomogram prediction of all-cause mortality

The C-index for the final analysis was used to estimate and compare different variables. log-rank test was used for comparison. Variables that achieved significant P < 0.05 were entered into the multivariable Cox regression analyses. A final model was selected using a backward step-down process [4, 5]. The final model included six predictors as follows, age, estimated glomerular filtration rate (eGFR), SBP, heart rate (HR), brain natriuretic peptide (BNP), and cardiac troponin I (TNI). Hazard ratios (HR) of the predictors for 1-year mortality were calculated and listed in Table 2.

**Table 2:** Univariable analysis and cox proportional hazards regression analysis.

**Variable**	**Univa-riable P**	**Multivariable Analysis**				**Selected Predictors for Building the Model**			
		**Hazard Ratio**	**95% CI**		**P value**	**Hazard Ratio**	**95% CI**		**P value**
			**lower limit**	**upper limit**			**lower limit**	**upper limit**	
Age, years	0.004				0.080				0.043
≤ 50		Reference							
50-80		0.256	0.058	1.137	0.073	0.239	0.054	1.046	0.057
> 80		0.588	0.327	1.057	0.076	0.572	0.323	1.013	0.056
eGFR, mL/min/1.73m^2^	< 0.001				0.008				0.002
< 30		Reference							
≥ 30		1.491	1.109	2.005	0.008	1.546	1.171	2.041	0.002
BNP,	< 0.001				< 0.001				< 0.001
≤1000		Reference							
1000-30000		0.127	0.045	0.359	< 0.001	0.115	0.044	0.303	< 0.001
> 30000		0.200	0.084	0.472	< 0.001	0.185	0.083	0.412	< 0.001
TNI,	< 0.001				< 0.001				< 0.001
≤ 1		Reference							
1-10		0.117	0.055	0.246	< 0.001	0.121	0.058	0.252	< 0.001
> 10		0.145	0.072	0.289	< 0.001	0.148	0.076	0.291	< 0.001
HR, bpm	< 0.001				< 0.001				< 0.001
≤100		Reference							
>100		0.325	0.195	0.542	< 0.001	0.326	0.196	0.542	< 0.001
SBP, mmHg	< 0.001				< 0.001				0.001
≤ 100		Reference							
100-180		10.163	1.257	82.191	0.030	9.114	1.162	71.498	0.035
> 180		2.935	0.394	21.865	0.293	2.720	0.371	19.937	0.325
WBC, 10^9^/L	0.084								
≤10									
>10									
LDL-C, mmol/L	0.914								
≤3.4									
>3.4									
Hemoglobin, g/L	0.002				0.926				
≤90		Reference							
>90		0.962	0.427	2.170	0.926				
Gender	0.389								
Hypertension	0.201								
Diabetes mellitus	0.047	1.083	0.632	1.857	0.772				
Current smoker	0.014	0.839	0.475	1.482	0.545				
Alcohol intake	0.701								

* HR: heart rate; SBP: systolic blood pressure; eGFR: estimated glomerular filtration rate (mL/min/1.73m^2^); BNP: B-type natriuretic peptide; TNI: troponin I; CI, confidence interval; WBC, white blood cell count; LDL-C, Low-density lipoprotein cholesterol.

**Figure 1:**
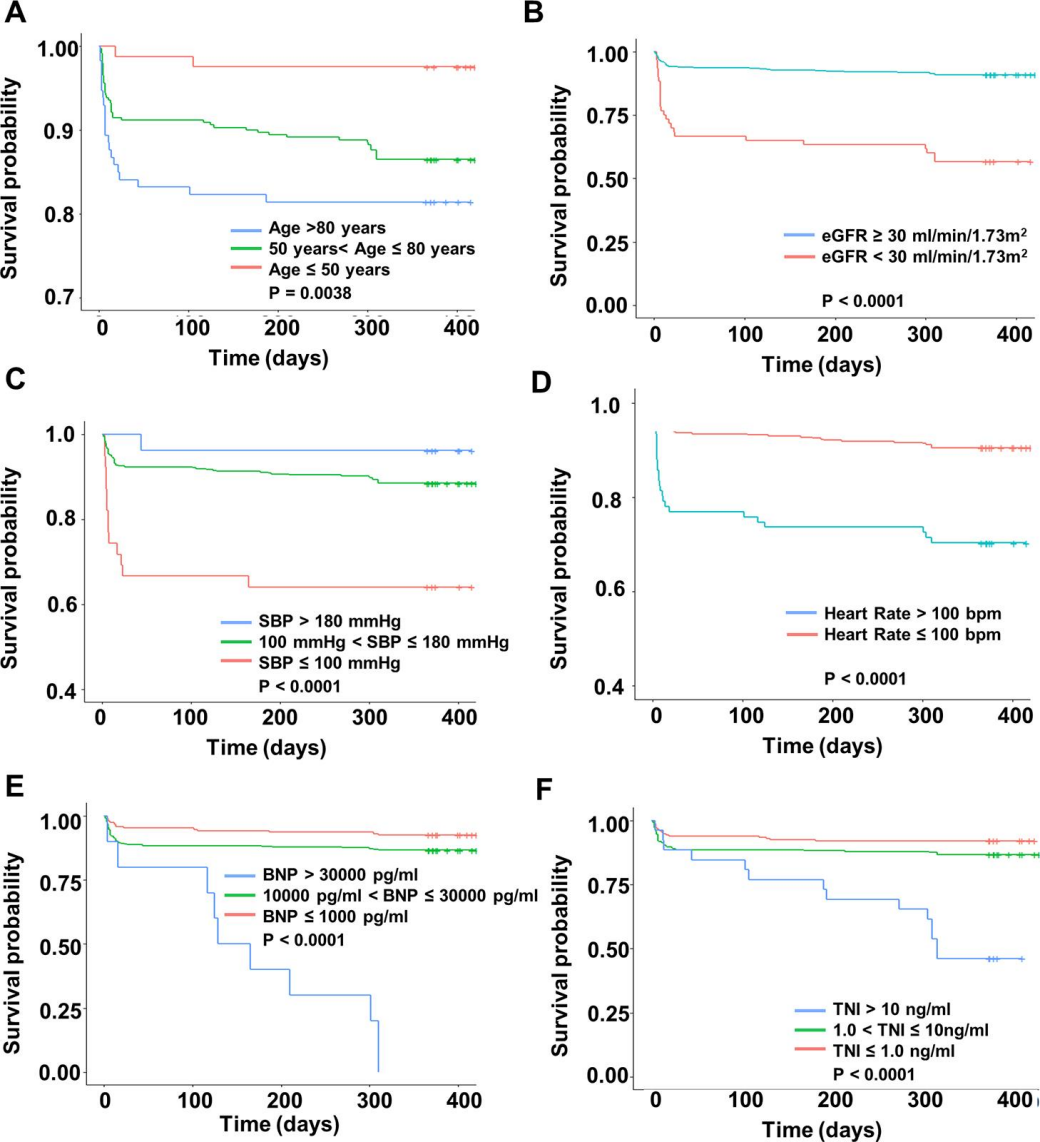
**Kaplan-Meier survival curves of 1-year survival according to six predictors.** The x-axis represents the time and the y-axis represents the overall survival in different subgroups. (**A**) age, (**B**) eGFR, (**C**) Systolic Blood Pressure, (**D**) heart rate, (**E**) BNP, and (**F**) Troponin I.

**Figure 2:**
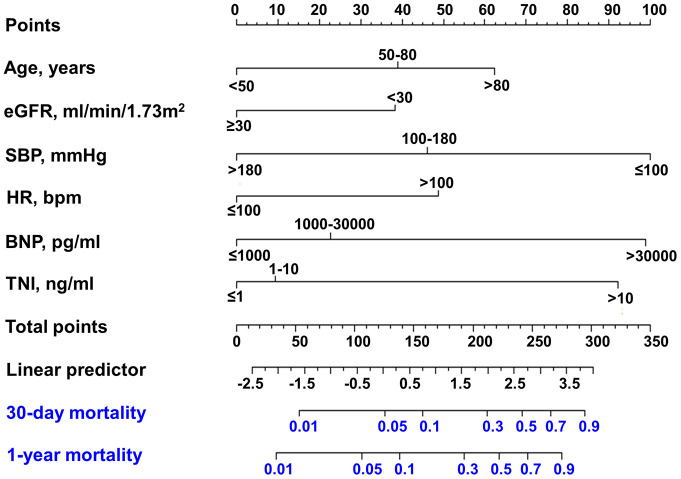
**Nomogram developed for predicting 30-day mortality and 1-year mortality.** Nomogram for 30-day mortality of AMI patients. The first row: point assignment of the variables; the second to seventh rows: six predictors; the eighth row: total points of six predictors; the ninth row: linear predictor; the tenth row: risk of 30-day mortality; and the eleventh row: risk of 1-year mortality.

Kaplan-Meier survival curves of 1-year mortality for each predictor are illustrated in Figure 1. The survival curves showed that there were significant differences in survival of each subgroup (all *P* < 0.05). Then, these variables were also used to create nomograms for estimating the probability of 30-day and 1-year mortality (Figure 2). The final nomogram model was named as Changzhou AMI (for short, C-AMI) model.

### Calibration and discrimination and of nomograms

**Figure 3:**
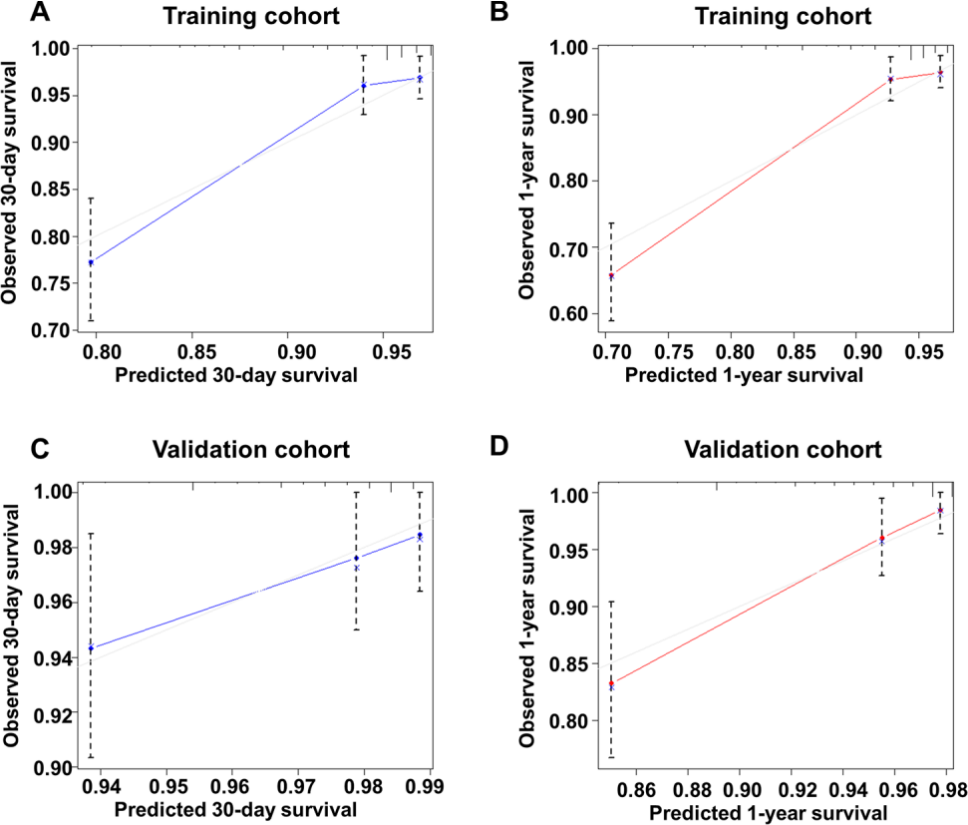
**Calibration curve of the new model for predicting 30-day and 1-year mortality in the training group and the validation group.** (**A**) Calibration curve of the new model for predicting 30-day in the training group. (**B**) Calibration curve of the new model for predicting 1-year in the training group. (**C**) Calibration curve of the new model for predicting 30-day in the validation group. (**D**) Calibration curve of the new model for predicting 1-year mortality in the validation group.

For nomogram model of 30-day mortality, calibration curves of the training group and the validation group were shown in Figure 3A and 3C. And for nomogram model of 1-year mortality, the calibration curves of the training cohort and the validation cohort were shown in Figure 3B and 3D. Together, these results suggest a good fit for our model.

**Figure 4:**
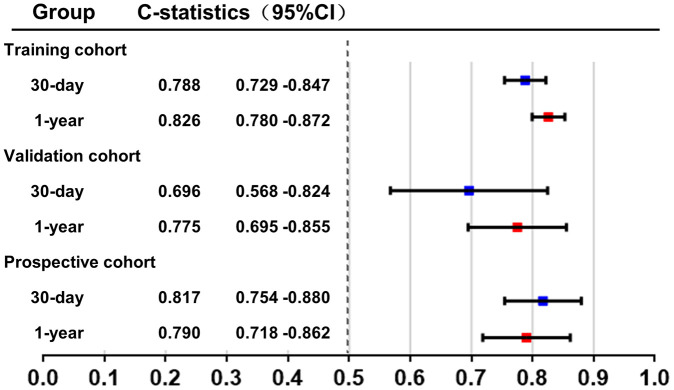
**C-index of the new model in the training group and the validation group.** C-index was calculated to evaluate the discrimination of the model and illustrated in this figure.

The C-index for the final 30-day mortality generated by bootstrap validations was 0.788 (95% CI: 0.729 - 0.847) in the training group and 0.696 (95% CI: 0.568 - 0.824) in the validation group. Moreover, the C-index for 1-year mortality in the training group and the validation cohort was 0.826 (95%CI: 0.780 - 0.872) and 0.775 (95%CI: 0.695 - 0.855), respectively (Figure 4). C-index of GRACE risk score for 30-day mortality was 0.762 (95% CI: 0.698 - 0.826) and for 1-year mortality was 0.722 (0.662 - 0.782) in training group. These results showed a good discrimination of the nomogram models.

### Comparisons between the risk predicted by the C-AMI model and that predicted by the GRACE score

**Figure 5:**
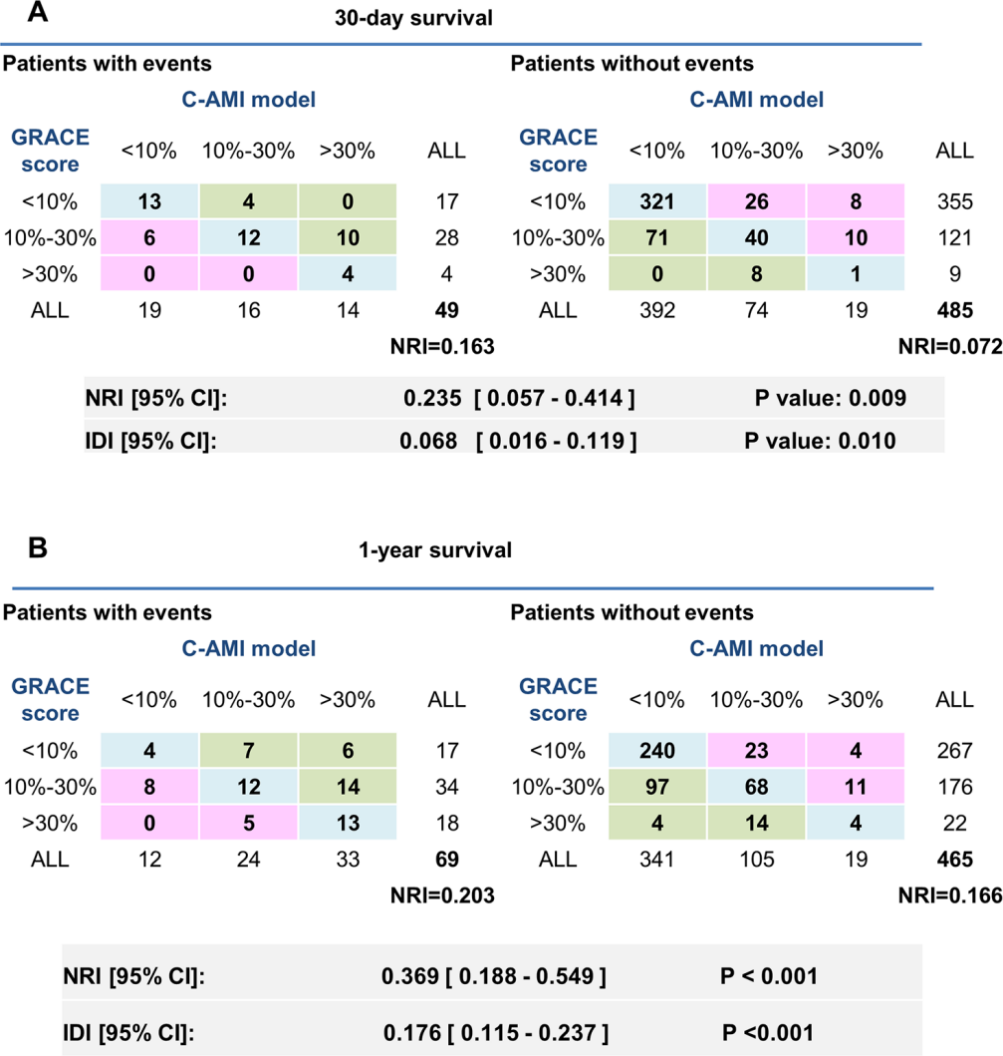
**Comparison in predicting 30-day and 1-year mortality between the C-AMI model and the GRACE score.** (**A**) NRI was calculated in the training cohort. We used 10% and 30% as thresholds to define patients at low (<10%), intermediate (10–30%), and high risk (>30%). IDI were also listed above. (**B**) NRI and IDI of the new model comparing with GRACE risk score for predicting 1-year mortality.

In the training cohort, we also calculated the GRACE score of each patient, and then made comparisons between the risk estimated by our C-AMI model and that by the GRACE score. First, net reclassification improvement (NRI) was calculated in the training cohort. Based on the previous studies [6], we used 10% and 30% as thresholds to define the risk grade of patients at low (<10%), intermediate (10 - 30%), and high risk (>30%), C-AMI model achieved an NRI of 23.5% as compared with the GRACE risk score (<10% as low risk, 10 - 30% as moderate risk, and 30% as highest risk) for predicting 30-day mortality (Figure 5A). In 49 patients with events within 30 days, 14 patients were correctly reclassified into a higher risk category by C-AMI model. On the other hand, 6 patients out of 49 were incorrectly reclassified to lower risk categories by GRACE risk score. Moreover, C-AMI model achieved an NRI of 36.9% as compared with the GRACE risk score for predicting 1-year mortality (Figure 5B). In 69 patients with events within 1 year, 27 patients out of 69 were correctly reclassified into a higher risk category by C-AMI model. On the other hand, 13 patients out of 69 were incorrectly reclassified to lower risk categories by GRACE risk score. The integrated discrimination improvement (IDI) are also listed in Figure 5. IDI showed a significant improvement in the accuracy generated by the C-AMI model when compared with the GRACE score. These results indicate that C-AMI model has better performance in predicting mortality than GRACE score.

**Figure 6:**
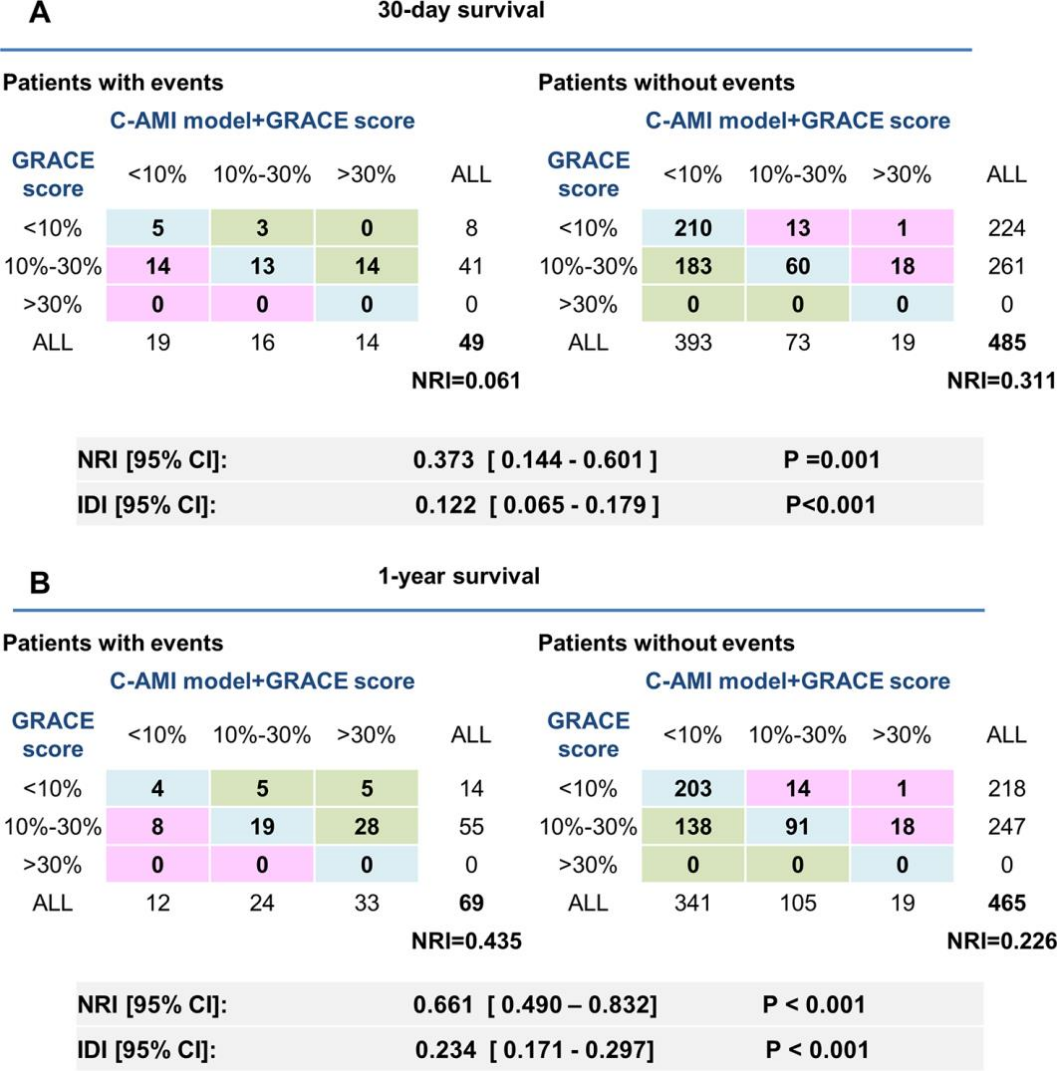
**Adjustment of the GRACE score by C-AMI model enables a more accurate appreciation of risk stratification in AMI patients.** (**A**, **B**) Reclassification ability of C-AMI model and GRACE score for 30-day (**A**) and 1-year mortality (**B**). NRI and IDI were calculated.

When C-AMI model was combined with GRACE risk score, the increase in prognostic power was paralleled by a huge increase in NRI (NRI for 30-day mortality: 37.3%; NRI for 1-years mortality: 66.1%) (Figure 6). These results were also confirmed by calculating IDI (12.2% for 30-day mortality; 23.4% for 1-year mortality). These results show that incorporating C-AMI model into GRACE risk score can significantly improve the predicting ability in patients with AMI.

### Sensitive analysis

**Figure 7:**
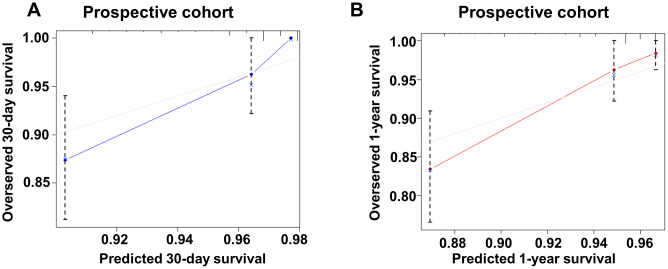
**Calibration curve of the new model for predicting 30-day and 1-year mortality in prospective cohort.** (**A**) Calibration curve of the new model for predicting 30-day in prospective group. (**B**) Calibration curve of the new model for predicting 1-year in prospective group.

A prospective cohort of 309 AMI patients (age 66.70 ± 14.43 years, 68.6% male) was used to further validate our nomogram model. The cohort was formed up with 159 patients from center-hospital area and 150 patients from yang-hu area. The C-index in this new cohort was 0.817 (95%CI: 0.754 - 0.880) for 30-day mortality and 0.790 (95%CI: 0.718 - 0.863) for 1-year mortality (Figure 4). These results confirm good discrimination of the model. The calibration curves of this new cohort were listed in Figure 7.

## DISCUSSION

The purpose of this investigation was to develop and verify a robust prognostic model to predict 30-day and 1-year mortality of AMI patients. We tested the hypothesis that the model developed herein would exceed the prediction fidelity of the GRACE score.

This investigation established a simple-to-use nomogram to predict 30-day and 1-year mortality in AMI patients. The nomogram was developed from 534 individuals and was validated as an illustrated model for predicting mortality in AMI patients. We also validated this new model in another prospective cohort. The results indicated that C-AMI model has good discrimination and calibration in the new cohort. Nomogram model was used for illustrating our model because nomogram is an useful tool of graphically representing the model into a graph through mathematical formula [7–10]. It is easy to calculate the risk of the occurrence of the final event by summation of the corresponding points of each variable. Thus, nomogram model is more intuitive and simpler, and is widely used in the prediction and evaluation of overall survival in patients. Meanwhile, clinicians can also quickly carry out risk stratification in the emergency room with the help of nomogram to identify patients with AMI at high risk of death. Thus, the nomogram of C-AMI model may serve as a valuable tool for clinical practice, providing up to 1-year risk counseling for AMI patients and guidance on preventive treatment for patients with a high risk of mortality. However, further clinical studies should be conducted to assess the effectiveness of preventive, therapeutic strategies based on this model.

AMI is a common severe disease, causing a tremendous medical burden on patients, their families, and the society. Early assessment of the prognosis of the disease is particularly critical [11, 12]. In this study, we constructed a predictive model for 30-day and 1-year mortality in AMI patients receiving interventional therapy. A nomogram was developed for clinicians to assess preoperatively short-term prognosis of the disease, and further provide guidance on early preventive measures. Our model included 6 variables: age, TNI, eGFR, SBP, heart rate, and BNP as primary predictors. These variables are easy to obtain in the early stage of admission, so that it is more convenient for clinicians to use and carry out early risk assessment.

Also, we compared our model with the GRACE score. The GRACE score includes eight predictors [3], including age, heart rate, blood pressure, serum creatinine level, Killip grade [13], pre-hospital cardiac arrest, ST segment depression, and increased myocardial enzymes. Granger et al [14] confirmed that the GRACE model had a good predictive value for mortality in patients with the acute coronary syndrome (ACS), which is commonly associated with three clinical manifestations: STEMI, non-ST elevation myocardial infarction (NSTEMI), or unstable angina. Consistent with the GRACE score, our prediction model showed that age, heart rate and SBP were independent predictors, being closely related to prognosis of AMI. In contrast, some variables were changed in our model. First, we included eGFR but not creatinine levels since the former reflects the renal function of patients more accurately without the influence of gender and weight. Second, Killip classification was not included in our model due to the presence of subjective bias. Third, we added BNP reflecting the cardiac function which was closely related to the prognosis of acute myocardial infarction [15]. Fourth, we included the level of TNI but not myocardial enzymogram as predictors because the level of TNI has greater specificity for myocardial injury. Finally, two risk factors included in the GRACE score (prehospital cardiac arrest and ST segment suppression) were not included in our model, as they did not improve the predictive value of our model and unnecessarily increased its complexity.

We found that there was a slight improvement between the C-AMI model and the GRACE scores in predicting 30-days mortality. But we found that our model appeared to be superior to the GRACE score in predicting 1-year mortality. Previous studies have reported that GRACE score combined with other biomarkers, such as GDF15, will further improve the accuracy of prediction ability [16]. We also found that when C-AMI model was combined with GRACE score, the value in risk stratification of AMI patients was significantly improved. The combination of the two models is more conducive to distinguish those high-risk AMI patients from the population, so as to take preventive measures in advance and reduce the mortality rate.

### Limitations

Our study has several limitations, mainly due to the characteristics of our database. Firstly, some new biomarkers for the prognosis of patients with AMI, such as growth differentiation factor (GDF-15) [17–20], high-sensitive troponin T, copeptin, soluble suppression of tumorigenicity 2(sST2) [18], were not taken into account in our model. Secondly, the model was developed and validated based on patients in two branches of our hospital, suggesting the presence of geographical limitations. In addition, it is necessary to confirm and modify our model in an independent external database with larger sample size. The model developed herein also only utilized six risk factors assessed at admission, which excludes the influence of other, potentially relevant factors. Thirdly, although our model calibrated well both in the development cohort and in two validation cohorts, the confidence intervals of lowest survival rate in predicted probability did not include the actual observed probability in both training and validation group. Finally, the dynamic change of some variables, especially biomarkers, in the course of the disease would lead to the change of important clinical information, influencing the prognostic evaluation. Therefore, how to integrate the dynamic change of variables into our model requires further investigation [21].

## CONCLUSIONS

In summary, this investigation developed a C-AMI model to predict short-term mortality in AMI patients. The model included six risk factors that are directly obtained and routinely collected in clinical practice. In the population utilized herein, the predictive value based on this C-AMI model was superior to the GRACE score. Moreover, due to fewer variables involved, our C-AMI model is a simple and easy-to-use means of predicting short-term prognosis in AMI patients.

## MATERIALS AND METHODS

### Ethical statements

Protocol approval was received from the institution review board at Changzhou No.2 People’s hospital. This study was approved by Changzhou No.2 People’s Hospital ethics committee. This Trial was registered in the Chinese clinical trials registry: ChiCTR1800014583. (http://www.chictr.org.cn/searchproj.aspx).

### Study population

The study population is partly based on Changzhou acute myocardial infarction registry (CZ-AMI) described before [22]. Briefly, CZ-AMI registry was a retrospective study of the risk of acute kidney injury in AMI patients from two single centers between 2013 and 2017. AMI was diagnosed according to the third universal definition of myocardial infarction [23].

Totally 1229 eligible patients were enrolled in this cohort study. C-AMI model was developed based on the training cohort (534 patients from a single center named center-hospital area) and tested it in the validation cohort (386 patients from another single center named yang-hu hospital area). Moreover, a prospective cohort of 309 patients were enrolled for further validation of the nomogram-based model. The study flow chart was included in supplementary information (Supplementary Figure 1).

### Data collection

The blood samples of hospitalized patients were collected and analyzed immediately at admission. Lab data (e.g. white blood cell count (WBC), the ratio of neutrophils, hemoglobin, cardiac troponin I (TnI), serum creatinine, brain natriuretic peptide (BNP) and so on) were collected. All biochemical measurements were carried out in standard laboratory techniques. The estimated glomerular filtration rate (eGFR) was calculated by the abbreviated MDRD equation according to the baseline serum creatinine concentration [24].

Basic clinic characteristics of enrolled patients were obtained by reviewing electronic medical records. The clinical variables such as age, gender, systolic blood pressure (SBP), diastolic blood pressure (DBP), heart rate, and a history of hypertension, diabetes mellitus, smoking, alcohol consumption and use of medication were documented.

### Coronary angiography and interventional therapy

Coronary angiography (CAG) or percutaneous coronary intervention (PCI) was conducted by experienced physicians in digital subtraction angiography (DSA) room. The procedural data were also collected.

### Follow-up and outcomes

The primary outcome of our study was 1-year all-cause mortality after AMI. The secondary outcome was 30-day all-cause mortality after AMI. Follow-ups were conducted by telephone, electronic medical record review and social security death index.

### Statistical analysis

Continuous variables are summarized as mean ± the standard deviation (SD) or medians and interquartile-range (IQR) and were compared using student t test or Kruskal-Wallis test. Categorical variables are summarized as counts and percentages and were compared using chi-square or the Fisher exact test, as appropriate. Kaplan-Meier survival analysis was used to show associations between each predictor and mortality. The multivariable time-to-event analysis was performed using Cox proportional hazards regression models. Hazard ratio (HR) and 95% Confidence interval [CI] were calculated.

Nomograms for predicting the risk of 30-day mortality and 1-year mortality were then established by Cox regression analysis [4, 25, 26]. Validation of the nomogram was assessed by discrimination and calibration. Harrell’s C-statistic [27] was calculated to evaluate the discrimination of our nomogram model. Calibration curve was used to evaluate the goodness of fit. Furthermore, net reclassification improvement (NRI) and integrated discrimination improvement (IDI) were calculated to evaluate the improvement of our new model when compared to the GRACE score.

All analyses were performed by SPSS (version 22.0, IBM Corp. Armonk, NY, USA) and R 3.4.3 (the R Core Team; 2017 R; a programming environment for data analysis and graphic). A *P*-value of less than 0.05 was considered statistically significant.

## Supplementary Material

Supplementary Figure 1
