# Supplementary material for: Nomograms based on pre-operative parametric for prediction of short-term mortality in acute myocardial infarction patients treated invasively
Source: Aging (Albany NY). 2020 Dec 11;13(2):2184–97. doi: 10.18632/aging.202230 (PMC7880403; doi:10.18632/aging.202230)
Supplement: Supplementary Figure 1 [file aging-13-202230-s001.pdf]

SUPPLEMENTARY FIGURE

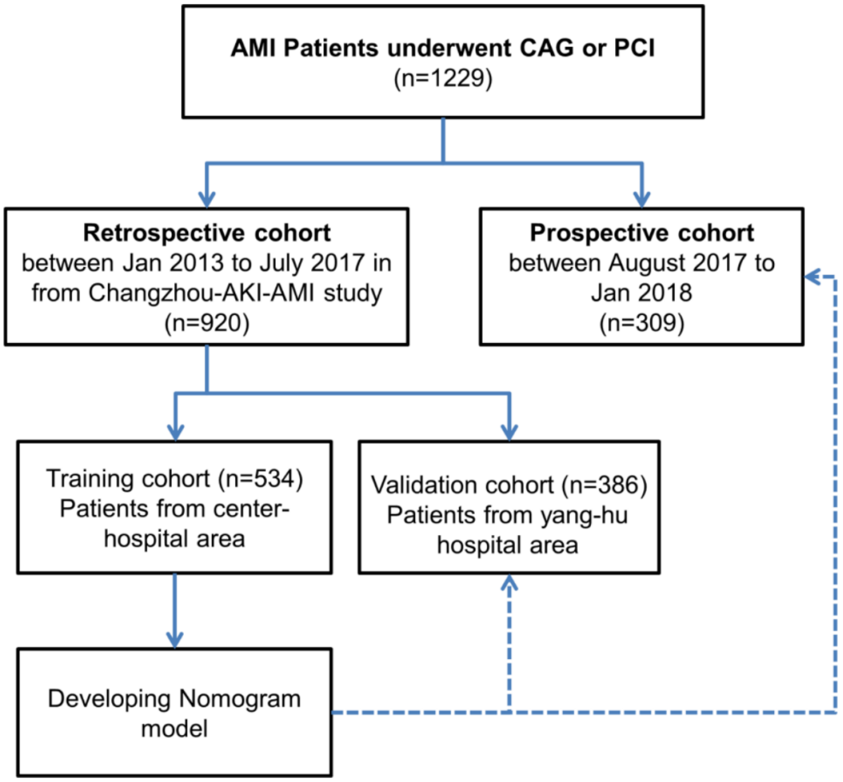

Supplementary Figure 1. Study flow chart. Totally 1229 eligible patients were enrolled in this cohort study. Model was developed with 534 patients from a single center and tested it in the validation cohort (386 patients from another single center named yang-hu hospital area). Moreover, a prospective cohort of 309 patients were enrolled for external validation. AMI = acute myocardial infraction; CAG = coronary angiography; PCI = percutaneous coronary intervention.
